# An Image Turing Test on Realistic Gastroscopy Images Generated by Using the Progressive Growing of Generative Adversarial Networks

**DOI:** 10.1007/s10278-023-00803-2

**Published:** 2023-03-13

**Authors:** Keewon Shin, Jung Su Lee, Ji Young Lee, Hyunsu Lee, Jeongseok Kim, Jeong-Sik Byeon, Hwoon-Yong Jung, Do Hoon Kim, Namkug Kim

**Affiliations:** 1 Biomedical Engineering Research Center, Asan Medical Center, Seoul, Republic of Korea; 2 Department of Gastroenterology, University of Ulsan College of Medicine, Asan Medical Center, Seoul, Republic of Korea; 3 Seoul Samsung Internal Medicine Clinic, Seoul, Republic of Korea; 4 Department of Health Screening and Promotion Center, University of Ulsan College of Medicine, Asan Medical Center, Seoul, Republic of Korea; 5 Department of Medical Informatics, Keimyung University School of Medicine, Daegu, Republic of Korea; 6 Department of Internal Medicine, Keimyung University School of Medicine, Daegu, Republic of Korea; 7 Department of Convergence Medicine, University of Ulsan College of Medicine & Asan Medical Center, Seoul, Republic of Korea

**Keywords:** Generative adversarial networks, Gastroscopy image, Synthetic image, Image Turing test, Progressive growing of generative adversarial networks

## Abstract

Generative adversarial networks (GAN) in medicine are valuable techniques for augmenting unbalanced rare data, anomaly detection, and avoiding patient privacy issues. However, there were limits to generating high-quality endoscopic images with various characteristics, such as peristalsis, viewpoints, light sources, and mucous patterns. This study used the progressive growing of GAN (PGGAN) within the normal distribution dataset to confirm the ability to generate high-quality gastrointestinal images and investigated what barriers PGGAN has to generate endoscopic images. We trained the PGGAN with 107,060 gastroscopy images from 4165 normal patients to generate highly realistic 512^2^ pixel-sized images. For the evaluation, visual Turing tests were conducted on 100 real and 100 synthetic images to distinguish the authenticity of images by 19 endoscopists. The endoscopists were divided into three groups based on their years of clinical experience for subgroup analysis. The overall accuracy, sensitivity, and specificity of the 19 endoscopist groups were 61.3%, 70.3%, and 52.4%, respectively. The mean accuracy of the three endoscopist groups was 62.4 [Group I], 59.8 [Group II], and 59.1% [Group III], which was not considered a significant difference. There were no statistically significant differences in the location of the stomach. However, the real images with the anatomical landmark pylorus had higher detection sensitivity. The images generated by PGGAN showed highly realistic depictions that were difficult to distinguish, regardless of their expertise as endoscopists. However, it was necessary to establish GANs that could better represent the rugal folds and mucous membrane texture.

**Supplementary Information:**

The online version contains supplementary material available at 10.1007/s10278-023-00803-2.

## Introduction

Gastrointestinal (GI) endoscopy is an essential tool for diagnosing and treating various GI diseases, including cancer which remains a global health burden and one of the important causes of cancer-related deaths worldwide [1, 2]. However, given the essential nature of this tool, it is still challenging to diagnose GI neoplasms by endoscopy [3, 4] because the detection and diagnosis depend on the endoscopist’s experience [5].

Several studies have recently reported the performance of deep learning-based artificial intelligence (AI) systems using endoscopic images to compensate for endoscopist experience. These reports have shown favorable performance for detecting GI neoplasms, predicting invasion depth, or classifying neoplasms [6–12]. The computer-aided detection and diagnosis (CAD) system for colonic neoplasms has well-established and proven clinical efficacy [13–15]. However, the CAD system for gastric neoplasms has demonstrated relatively poor performance compared to colonic neoplasms [16]. The reason is that the background mucosa of gastric neoplasms accompanies chronic inflammation, such as in atrophic gastritis and intestinal metaplasia [11, 16]. Given the sensitivity of deep learning to background noise, these studies have significant limitations. They needed a large dataset of real-world and high-quality images. Such images have been associated with high costs, increased time, and ethical issues such as privacy concerns [17].

In research to overcome the vulnerability of deep learning to background noise or adversarial samples, Goodfellow et al. [18] introduced generative adversarial networks (GANs), a neural network to generate the realistic distribution of the training dataset. GAN is a model suitable for medical imaging research that can collect a large number of normal data through health checkups. In particular, the possibility of detecting abnormalities using GAN has been dramatically expanded, and many studies on abnormality detection using GAN have been reported in the medical field [19–24]. However, high-resolution and highly represented endoscopic images must be generated to obtain high-sensitivity screening performance. Many previous studies employing GANs in endoscopic images have focused on their application to low-quality images due to the limited performance of the image-generating models. However, we have focused our research on the high image-generating ability of the endoscope to build a more sensitive screening of CAD from a disease detection perspective.

Progressive growing of the GAN (PGGAN) [25] is one of the GAN that can create high-definition and high-quality images. This network has been applied in image processing and the medical field rather than matching endoscopic camera resolution. Park et al. reported that PGGAN could synthesize high-resolution body CT images, proven using the visual Turing test [26]. The result of the Turing test found a lack of accuracy in the thoracic abdominal junction and anatomical details. We speculated that PGGAN would have problems generating specific structures in the same way on endoscopic imaging.

**Fig. 1:**
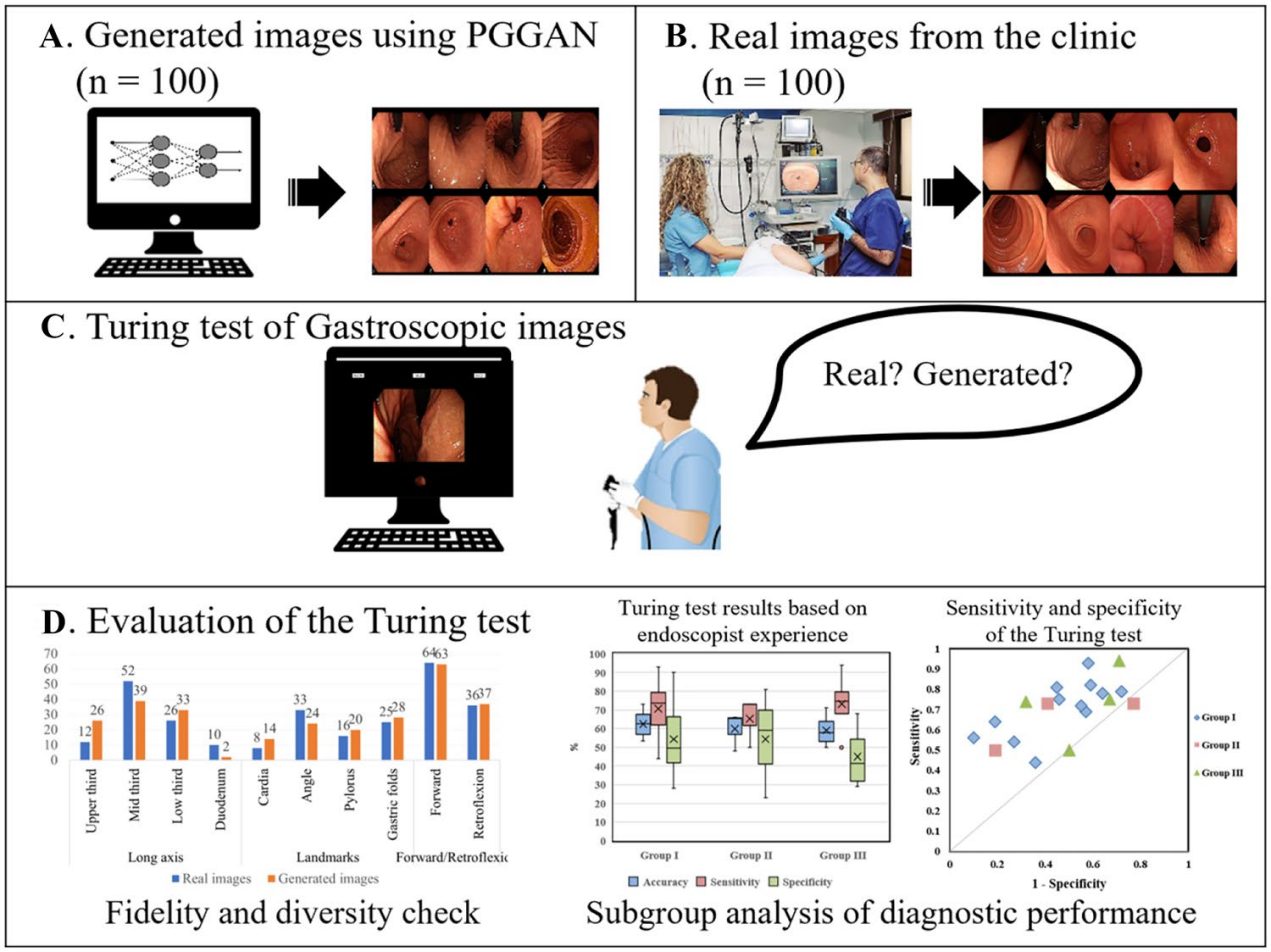
Process of gastric endoscopy image Turing test, **A** randomly generated 100 gastroscopy images using Progressive growing of GAN (PGGAN), **B** randomly extracted 100 images from health checkup data, **C** Turing test on 19 endoscopists to classify 100 normal images and 100 synthetic images, **D** Analyzing the diagnostic results of the Turing test

As described above, in the field of endoscopy, various studies using GAN, such as lesion synthesis and abnormality detection techniques, have been attempted. However, most of the research has been done with low-quality images. A GAN that produces low-quality images does not very well reflect small polyps or mucosal patterns. For this reason, we tried to analyze why it is challenging to generate an endoscopic image of high quality. Therefore, we employed a PGGAN to generate highly realistic-looking gastroscopy images and performed a visual Turing test as the first step towards a GANs-based AI system endoscopy. The overall process of our visual Turing test of the gastroscopic images was summarized in Fig. 1.

## Methods

### Data Acquisition

Electronic medical records were retrospectively reviewed to find patients who underwent gastroscopy in the Health Screening and Promotion Center of Asan Medical Center. A total of 166,997 images from 4,165 normal patients were obtained. However, various artifacts were existed in endoscopic imaging, such as motion-blur and light reflections. Also, both the endoscopic images of the gastric cardia and the endoscope itself in the retroflexion view were excluded, as the endoscope's distortion in the generated image is frequently identified as an abnormal structure. Since such artifacts may interfere with the learning representation of gastric endoscopy, 1000 images containing artifacts were selected manually and trained using supervised learning techniques to differentiate them. Data cleansing will be described in more detail in the next Data cleansing session. In this manner, 59,937 images were cleansed as there were motion-blurred or color-blurred images in the captured video. Finally, 107,060 images were used for training. Figure S1 demonstrated a schematic diagram of the process of collecting the training dataset by extracting an endoscopic image; the images were upsampled from 350^2^ pixels to 512^2^ pixel-sized images using OpenCV’s resize transform with third-order spline interpolation. This is since a ${2}^{n}$ sized image is required to train the PGGAN generator.

PGGAN was used to generate high-resolution gastroscopic images up to 512^2^ pixels, generated by applying the progressive growth technique to the generator. We used an official implementation code of PGGAN in this study. Since the training of PGGAN proceeds as the size of the input image of n power 2 gradually increases, the training image size gradually increased from 4^2^ to 512^2^ pixels, and the batch size decreased from 512 to 16, respectively, as the computational load increased with increasing resolution.

**Fig. 2:**
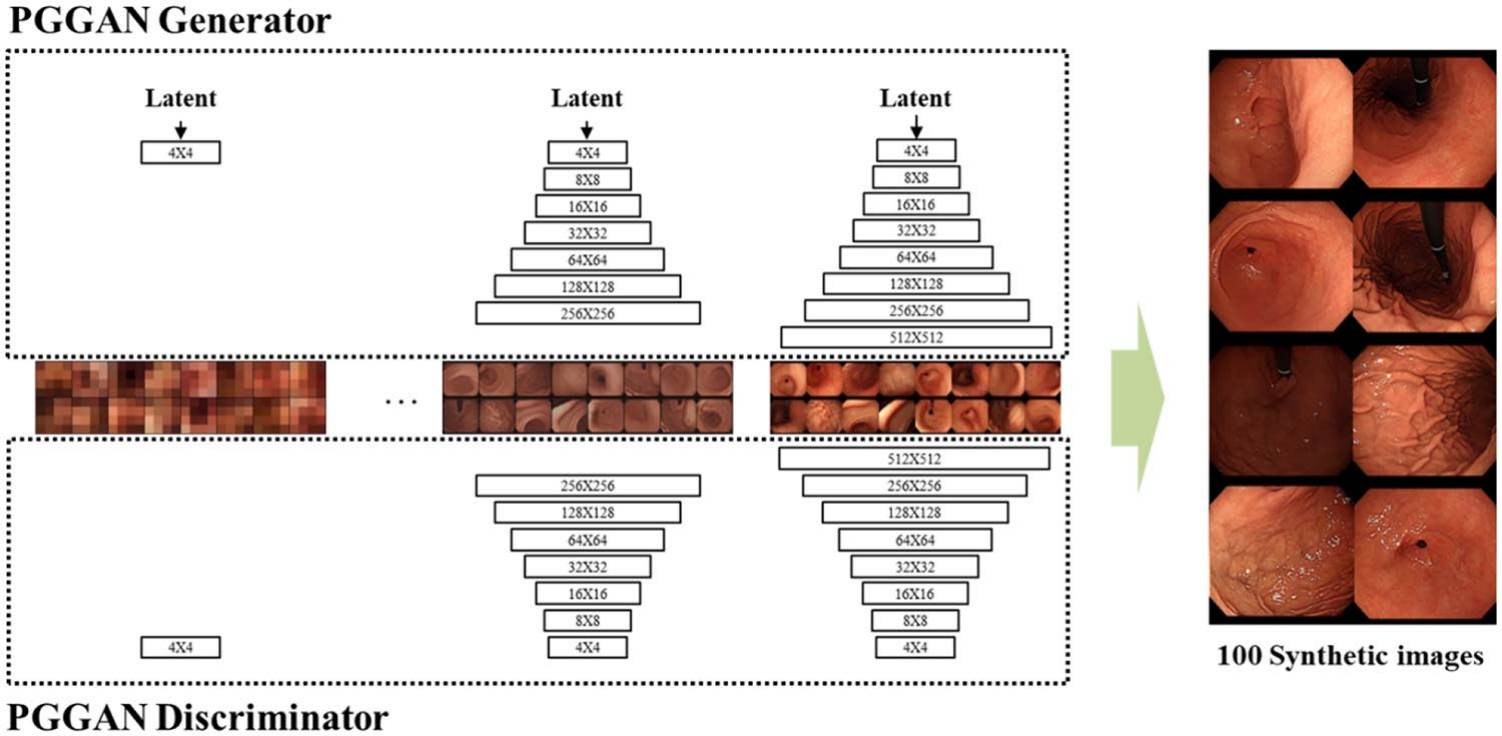
The training process of Progressive growing of GAN (PGGAN). As the training continues, the generator and discriminator increase the resolution of generated images to 512^2^ pixels. There are 8 sample endoscopic images generated using progressive growing at 512^2^ pixels on the right

The learning rate of PGGAN was set to 1e-3 while training. Figure 2 shows the process of PGGAN’s step-by-step generation of 100 images for the Turing test. The PGGAN training assessment was based on Fréchet inception distance(FID) [27] scores and expert visual inspection in every 4000 images on the PGGAN. During training PGGAN, the FID score was converged at an average of 6.0 after 6 million images were shown. Finally, the training weight was selected according to the visualized verification result of one expert who was excluded from the Turing test. The training took 5 days with two NVIDIA Titan RTX GPUs.

### Data Cleansing

We discovered that the acquired normal endoscopic images contained various artifacts. Assuming that low-quality original images would result in low-quality generated images, we aimed to exclude artifact-contained images. Since it would require a significant effort to examine the quality of a total of 166,997 images, we planned to construct a small image quality dataset to perform data cleansing. We annotated 1000 high-quality and low-quality endoscopic images each from our real dataset. These images were randomly split into training, validation, and testing sets in a 6:2:2 ratio. We used a ResNet18 [28] model pre-trained on ImageNet to train a binary classification model, which achieved an accuracy of 95% in the testing dataset. We applied this model to the remaining 164,997 images to cleanse the dataset, a total of 59,937 images were cleansed using ResNet18.

### Image Turing Test on the Realism of Synthetic Endoscopy Images

The validation set consisted of 200 gastroscopy images (100 synthetic images and 100 real images). All synthetic images in the validation set were automatically generated by our modified PGGAN model and were not individually selected by the researchers to avoid selection bias. The real images were taken at the Asan Medical Center, which did not otherwise participate in the realism assessment study. A website was created to upload the validation set, with the posting and displaying of the 200 images being performed randomly as Figure S2. Nineteen endoscopists (twelve endoscopists with < 5 years of experience [Group I]; three endoscopists with 5–10 years of experience [Group II]; and four endoscopists with > 10 years of experience [Group III]) independently evaluated each of the 200 images, and visually decide on whether each image was real or fake with no time limit and no prior information on the number of actual or fake images. To investigate the features of obviously artificial images, we defined artificial images as those synthetic images that the majority of readers (more than ten readers) decided were fake.

### Statistical Analysis

The mean accuracy, sensitivity, and specificity of the 19 readers were calculated. Sensitivity is the probability of detecting real images among the real images, and specificity is the proportion of identification of generated images. General estimation equations were used to compare the accuracy, sensitivity, and specificity across the reader groups with different experience levels (Group I, Group II, and Group III), and across the anatomical subgroups. Since there were only 19 participants in our Turing test, a nonparametric Kruskal–Wallis test [29] was performed to analyze whether the difference between the three groups of the independent variable level was significant. The significance level was corrected for multiple comparisons using the Bonferroni-holm correction [30], and inter-reader agreement was evaluated using the intraclass correlation coefficient [31]. The R version 3.6.3 was used for statistical analysis, with a significance level of *P* < .05.

## Results

### Overall Assessment of the Visual Turing Test

**Fig. 3:**
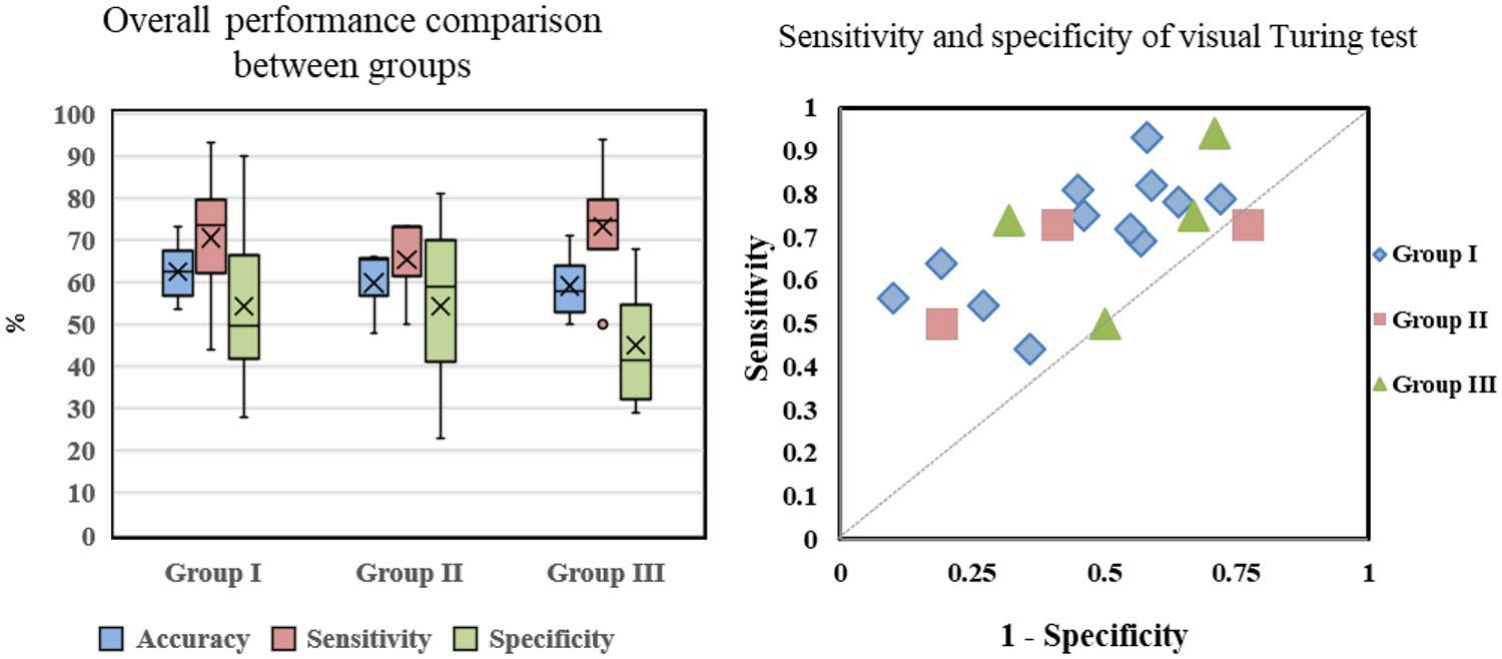
**Left** Result of differentiating performance difference between the three groups, **Right** Sensitivity and specificity of visual Turing test of synthesized high-resolution gastroscopy. There is no significant difference in discriminating between the three groups in the above two images

The mean accuracy, sensitivity, and specificity of the 19 endoscopists were 61.3%, 70.3%, and 52.4%, respectively. Table 1 and Fig. 3 summarize the results of the realistic assessment of all images by 19 endoscopists with different experience levels (Group I: 62.5, Group II: 59.8, and Group III: 59.1; *P* > .05). There was no correlation between endoscopist groups since the standard deviation of mean sensitivity and mean specificity was tremendous. The average sensitivity tended to be about 17.9% higher than the average specificity.

**Table 1:** Realism assessment of all images by 19 endoscopists (E01–E19)

**Groups readers**	**Accuracy (%)**	**Sensitivity (%)**	**Specificity (%)**
**Group I (*n* = 12)**	62.5 ± 6.8	70.6 ± 13.9	54.3 ± 19.1
E 01	64.5	75.0	54.0
E 02	63.5	54.0	73.0
E 03	56.0	69.0	43.0
E 04	58.5	72.0	45.0
E 05	72.5	64.0	81.0
E 06	54.0	44.0	64.0
E 07	61.5	82.0	41.0
E 08	73.0	56.0	90.0
E 09	53.5	79.0	28.0
E 10	68.0	81.0	55.0
E 11	57.0	78.0	36.0
E 12	67.5	93.0	42.0
**Group II (*n* = 3)**	59.8 ± 10.3	73.2 ± 18.0	54.3 ± 29.3
E 13	66.0	73.0	59.0
E 14	48.0	73.0	23.0
E 15	65.5	50.0	81.0
**Group III (*n* = 4)**	59.1 ± 9.2	65.3 ± 13.3	45.0 ± 29.3
E 16	61.5	94.0	29.0
E 17	71.0	74.0	68.0
E 18	54.0	75.0	33.0
E 19	50.0	50.0	50.0
Average (All)	61.3 ± 7.5	70.3 ± 14.1	52.4 ± 17.8
Kruskal–Wallis chi-squared (H)	0.64	0.99	0.64
Overall *P*	0.73	0.61	0.73

Group I endoscopists with < 5 years of experience, Group II endoscopists with 5–10 years of experience, and Group III endoscopists with > 10 years of experience

### Subgroup Analysis According to the Location

**Fig. 4:**
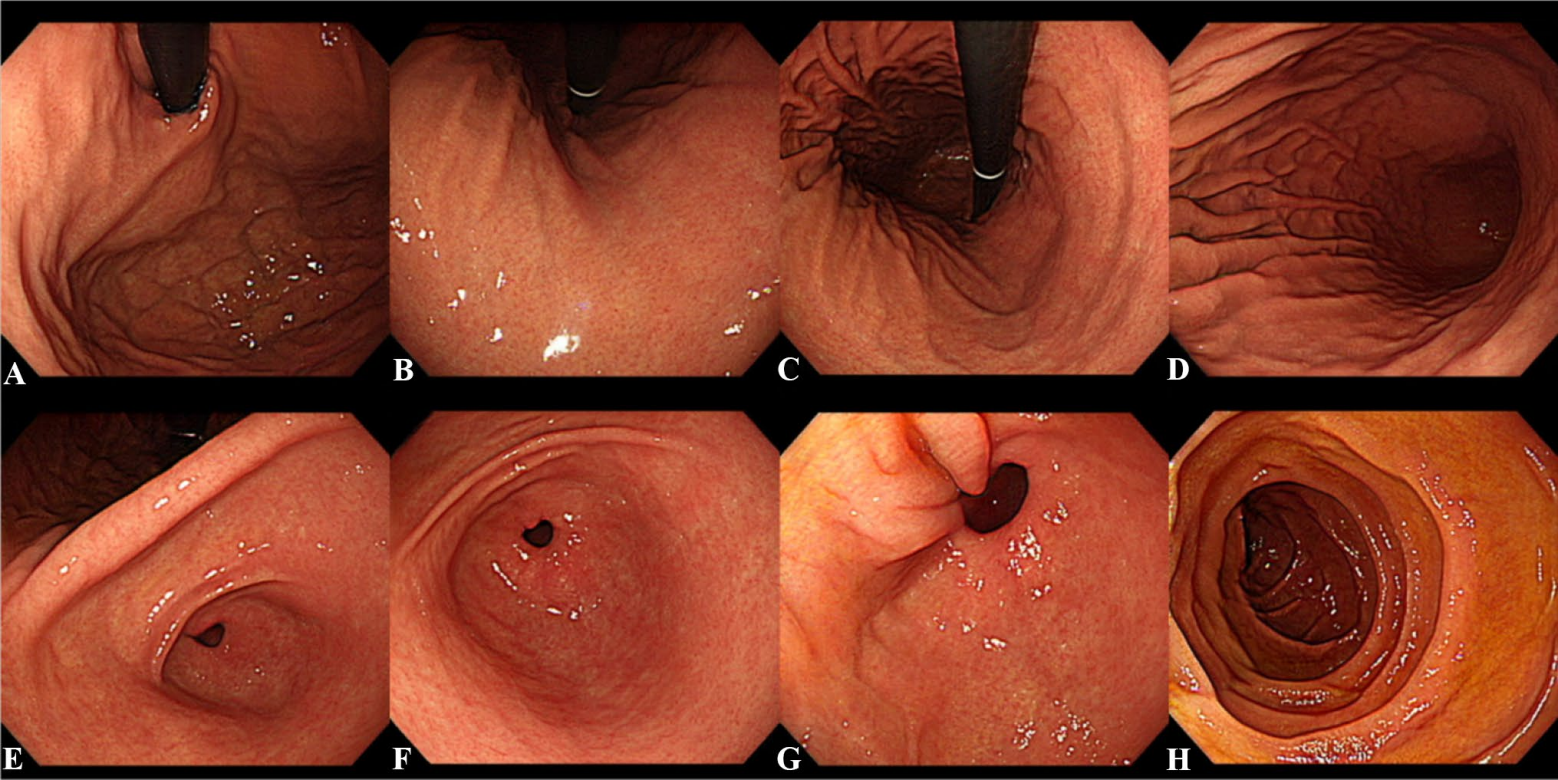
Sample images of synthesized high-resolution gastroscopy. **A** Cardia and fundus in retroflexion view. **B** Lesser curvature and anterior wall of a high body in retroflexion view. **C** High body and mid body in retroflexion view. **D** Mid body and low body in forward view. **E** Angle and antrum in retroflexion view. **F** Antrum and pylorus in forward view. **G** Pylorus in forward view. **H** 2nd portion of duodenum in forward view)

**Fig. 5:**
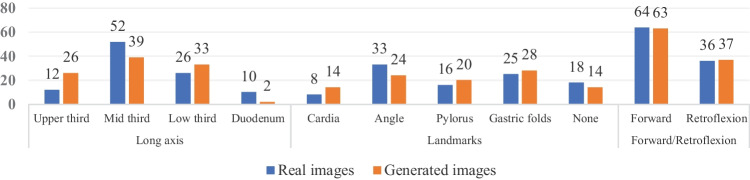
Comparison of the number of images generated for each part of the stomach. (Total 100 generated images) The gastroscopy images were randomly generated without adjusting the distribution

To analyze whether PGGAN generates images for each region of the stomach and duodenum, subgroup analysis by location was conducted. Figure 4 shows sample endoscopic images generated by PGGAN for each part of the stomach and duodenum. The number of images for each part of the 100 randomly selected real images and generated images were shown in Fig. 5. Although the distribution of the generated image is not precisely the same, it was confident that the generated image with a trend similar to the actual distribution. Table 2 shows the analysis results for each anatomical location. As there was no difference in distinguishing the actual gastroscopy image and the fake image according to experience, we analyzed only the differences for each region. There was no significant difference in diagnostic performance according to the anatomical location and endoscopic view. However, there was a statistically significant difference in sensitivity according to anatomical landmarks; the sensitivity to the pylorus was higher (*P* = .002).

**Table 2:** Subgroup analysis of diagnostic performance with respect to the anatomical location

**Longitudinal axis images**: upper third, middle third, lower third of the stomach, and duodenum				
**Type**	**Accuracy**	**Sensitivity**		**Specificity**
Upper	65.1 ± 8.5	73.7 ± 13.3		44 ± 20.6
Middle	59.7 ± 8.3	64.6 ± 17.6		56.5 ± 19.6
Lower	61.8 ± 9.9	72.8 ± 15.6		48.6 ± 24.7
Duodenum	59.9 ± 15.1	78.9 ± 26.2		48 ± 26.4
Kruskal–Wallis chi-squared	2.84	6.68		2.79
Overall *P*	0.42	0.08		0.43
**Anatomical landmarks:** cardia, angle, pylorus, gastric folds				
**Type**	**Accuracy**	**Sensitivity**	**Specificity**	
cardia	64.1 ± 9.8	72.6 ± 15.5	45.9 ± 27.6	
angle	57.5 ± 10.6	64.6 ± 16.0	52.8 ± 21.9	
pylorus	63.5 ± 12.1	**81.2** ± **17.2**	47.7 ± 24.7	
gastric folds	59.8 ± 8.0	64.3 ± 17.0		55.1 ± 19.9
Kruskal–Wallis chi-squared	3.79	**14.96**		1.31
Overall *P*	0.29	0.002**		0.73
**Forward and retroflexion view**				
**Type**	**Accuracy**	**Sensitivity**		**Specificity**
Forward	59.8 ± 9.4	65.7 ± 19.4		53.9 ± 21.7
Retroflexion	62.5 ± 7.3	70.8 ± 12.8		53.1 ± 17.8
Kruskal–Wallis chi-squared	0.646	1.334		0.0002
Overall *P*	0.421	0.248		0.988

*P < 0.05; **P < 0.01

### Inter-Observer Agreement in the Synthetic and Real Images

Intraclass correlation coefficients were used to analyze the agreement between the three groups to identify generated images. The interobserver agreement for all endoscopists was observed to be 0.74 for the entire image set, as shown in Table 3. However, when subgroup analysis was performed according to clinical endoscopic experience, Group II and Group III showed poor average Intraclass correlation coefficients below 0.3.

**Table 3:** Intraclass correlation coefficients of inter-reader agreement to identify fake images

Groups	Subject	Average intraclass correlation	95% Confidence interval
Group I	12	0.7442	0.6634 to 0.8124
Group II	3	0.2586	−0.03422 to 0.4790
Group III	4	0.1052	−0.2199 to 0.3608
Total	19	0.7430	0.6638 to 0.8108

### Analysis of the Features of Obviously Artificial Images

**Fig. 6:**
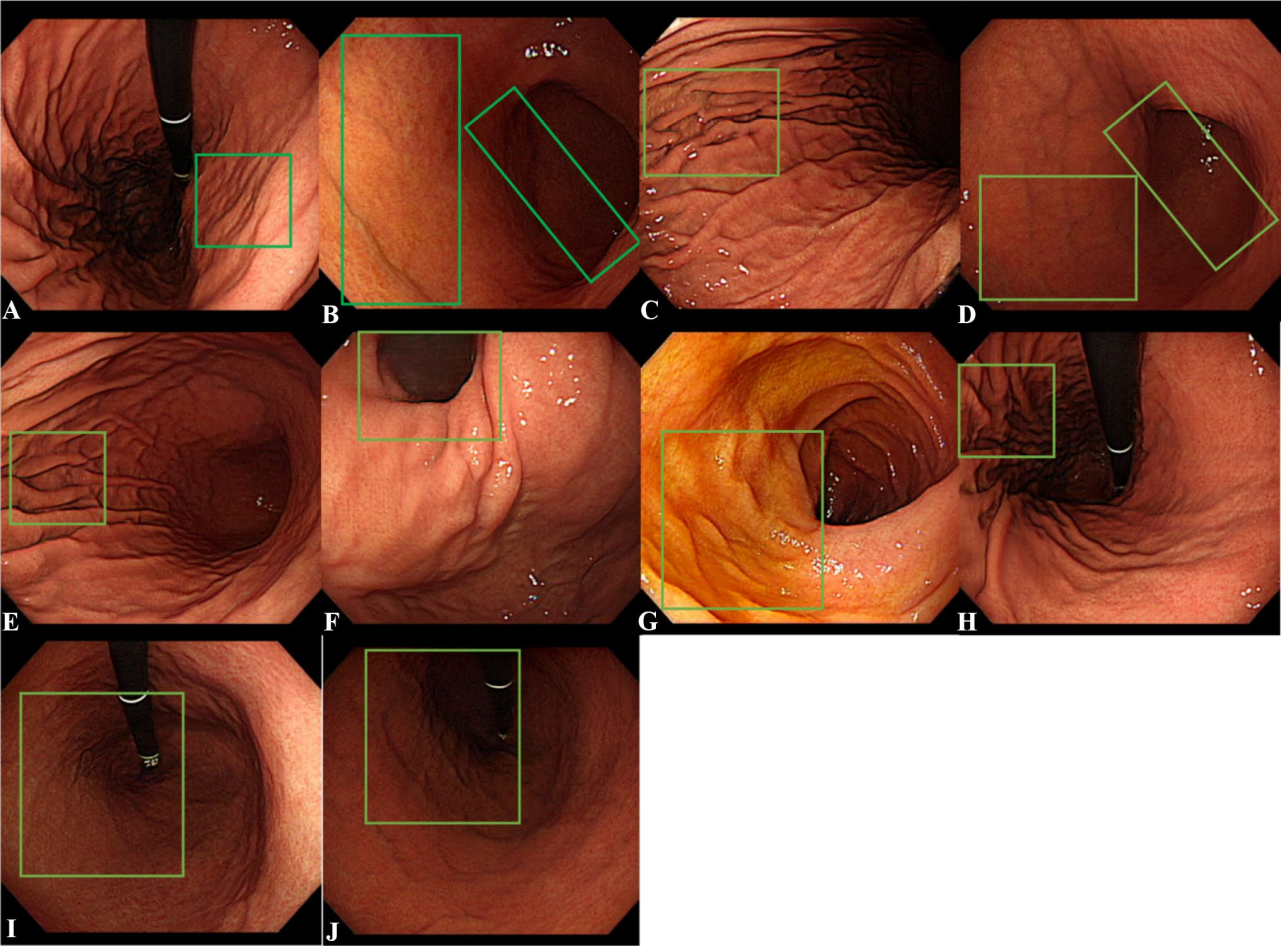
Synthetic images that the majority of readers decided were fake. **A** Irregular transverse folds in the lesser curvature. **B** Absence of normal gastric folds on the greater curvature of the lower body and scattered hyperemic mucosa and asymmetry between anterior wall and posterior wall of the angle. **C** Abrupt discontinuation and fusion of gastric folds in the greater curvature. **D** Absence of normal gastric folds on the greater curvature of the low body and asymmetry between anterior wall and posterior wall of the angle. **E** Abrupt discontinuation of gastric folds in the greater curvature. **F** Absence of an endoscope in the esophagogastric junction. **G** Discontinuation of circular folds on the duodenum. **H** Abrupt discontinuation and fusion of gastric folds in the greater curvature. **I** Absence of gastric folds and fundus. **J** Absence of gastric folds and fundus)

One hundred of the randomly chosen synthetic images were included in the image Turing test. The mean number of endoscopists who correctly answered fake for each synthetic image was 9.95 ± 3.08 (CI 95%, 9.34–10.56). Ten of the synthetic images were detected as fake by more than 10 out of 19 endoscopists. One experienced endoscopist reported the key features used to detect the images as fake for these synthetic images. The key features of 10 synthetic images were described in Fig. 6; overall, they showed uneven structure and an absence of typical structure.

## Discussion

In this study, we used the PGGAN model to generate realistic gastroscopy images and evaluated the realism of the images generated by 19 endoscopy physicians with the image Turing test. In the Turing test results, 19 endoscopists generally identified fake images with sensitivity and less specificity. Accuracy does not make a meaningful distinction between real and fake images. Also, in a subgroup analysis of endoscopic experience, there was no significant difference in the discrimination between real and synthetic endoscopic images.

Recently, many health experts are conducting regular checkups, and developments in endoscopic data storage have made it easy to access common imaging datasets to many researchers and clinicians. Normal and abnormal endoscopic images can, therefore, be taken frequently; however, normal images are more effective and useful for collecting huge datasets than abnormal images since they are easier to curate and provide more data. To date, supervised deep learning-based models have been successfully applied in the detection and classification of specific diseases in various medical fields [32]. However, deep learning techniques may be challenged in endoscopy due to the large range of normal variations and non-neoplastic conditions such as inflammation. Neoplastic lesions can be disregarded or unnoticed during the screening endoscopy, leading to a missed diagnosis of both the index lesion and any simultaneous lesions [3, 4, 33]. Therefore, meticulous inspection through the deep learning-based AI system for gastroscopy may improve diagnostic performance.

We believe that creating these highly realistic images may be the first step in applying GAN to the development of models applicable in the medical fields described above. This is the first study that systematically evaluated the realism of GAN-generated synthetic gastroscopy images through the intuition of numerous endoscopists. Therefore, the realistic nature of the synthetic images used in the previous studies was not confirmed. In addition, most previous studies focused on improving deep learning-based task performance resulting from the generation of images with diseases, using synthetic abnormal images to adjust for data imbalances; they did not consider the generation of normal images.

Recently, several studies have shown the favorable performance of deep learning-based AI systems in endoscopy and there were promising results in colonoscopy. However, AI systems for the detection of gastric neoplasms have exhibited poor performance compared to that of colonic neoplasms. In a previous study, a real-time AI system using white light endoscopic images showed high sensitivity (94.2%) similar to expert endoscopists in detecting upper GI cancer; however, the positive predictive value of the AI system (81.4%) was relatively lower than that of experts (93.2%) [8]. Hirasawa et al. also reported a low positive predictive value (30.6%) in deep learning-based AI systems for gastric cancer [16]. The causes of false positives by AI systems in the study included benign lesions such as gastritis, atrophy, or intestinal metaplasia and normal structures such as the gastric angle, pylorus, amounts of mucus, and elevation of gastric wall during peristalsis. Our PGGAN could create realistic gastric mucosa and a specific typical structure, including the gastric angle, pylorus, and the endoscope itself. The detection of gastric neoplasms using GANs will help to improve performance by reducing these errors. A previous deep learning based-AI study attempted a binary classification of gastric ulcers and gastric cancer but showed lower accuracy (77.1%) [34]. This performance is possible because they used a relatively smaller number of cases (220 benign ulcer images and 367 cancer images) than other deep learning-based studies. The need for a large dataset of real-world and high-quality images is one of the main limitations of deep learning. The multi-institutional data sharing and research agreements through efforts to address ethical issues in medical AI research are important for obtaining a large dataset of real-world and high-quality images. Also, the development of GAN can increase data quantity and quality with relatively few ethical issues and low costs. Our pre-trained GAN for healthy gastroscopy images will be helpful to investigate related research.

Our study has some limitations. First, the visual Turing test was conducted using 512^2^ resolution images. Recently, since clinical endoscopists deal with full high-definition endoscopic images, it is necessary to evaluate them in higher-quality images. The future work will increase GAN output resolution further to match technical advances. Secondly, the sensitivity according to anatomical landmarks was statistically significant in the subgroup analysis, specifically for the pylorus. We did not give endoscopists any prior information on the number of actual or fake images in the image Turing test for a precise validation test. Nineteen endoscopists tended to indicate the test image as real, as it was difficult to distinguish real or fake images. As a result, thirteen out of nineteen endoscopists had higher sensitivity than specificity. The specificity according to anatomical landmarks showed a low tendency (45.9–55.1%). Third, our evaluation was not performed with various modern generative networks other than PGGAN. We chose PGGAN as it was the most recent state-of-the-art at the time of our Turing Test analysis. We should determine if we can overcome the lack of generative performance by employing cutting-edge networks that are superior to PGGAN at generating high-quality images. Finally, to test PGGAN's capacity to generate realistic images, real endoscopic images with color jittering and motion blur were removed. Since many low-quality images were included in the actual endoscopic image, data bias may have been introduced while removing them.

In conclusion, our PGGAN model generated highly realistic endoscopic images of the stomach and duodenum, difficult to distinguish regardless of endoscopist experience. Since this PGGAN model can produce high-quality endoscopic images, it may help mitigate the limitations of deep learning, including class imbalances, challenges of data-sharing, and privacy concerns. In addition, if the expression of folds and texture, which the PGGAN lacks, is improved, it may be sufficient to detect abnormal lesions of the upper GI.

## Supplementary Information

Below is the link to the electronic supplementary material.

Supplementary file1 (DOCX 1078 KB)

## Data Availability

The datasets are not publicly available due to restrictions in the data-sharing agreements with the data sources. Ethics approval for using these de-identified slides in this study was granted by the Institutional Review Board of Asan Medical Center.
