# Supplementary material for: An Image Turing Test on Realistic Gastroscopy Images Generated by Using the Progressive Growing of Generative Adversarial Networks
Source: J Digit Imaging. 2023 Mar 13;36(4):1760–9. doi: 10.1007/s10278-023-00803-2 (PMC10406771; doi:10.1007/s10278-023-00803-2)
Supplement: Supplementary file 1 — Supplementary file1 (DOCX 1078 KB) [file 10278_2023_803_MOESM1_ESM.docx]

**Supplementary Appendix I**


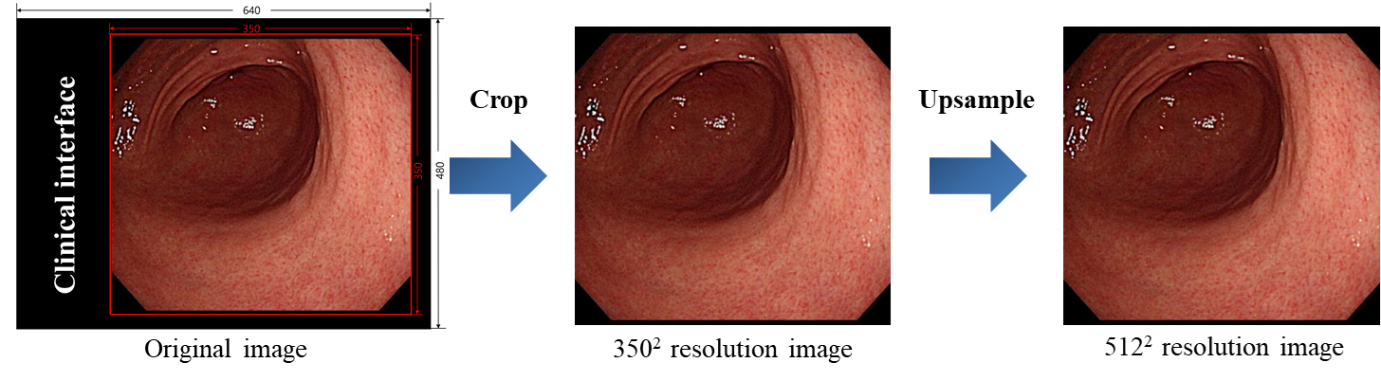


**Figure S1.** Data acquisition process from endoscopy raw images on the clinical setting diagnosis interface screen, only the endoscopic image (350^2^ pixel-sized) was extracted and unsampled by 512^2^ resolution image.

**
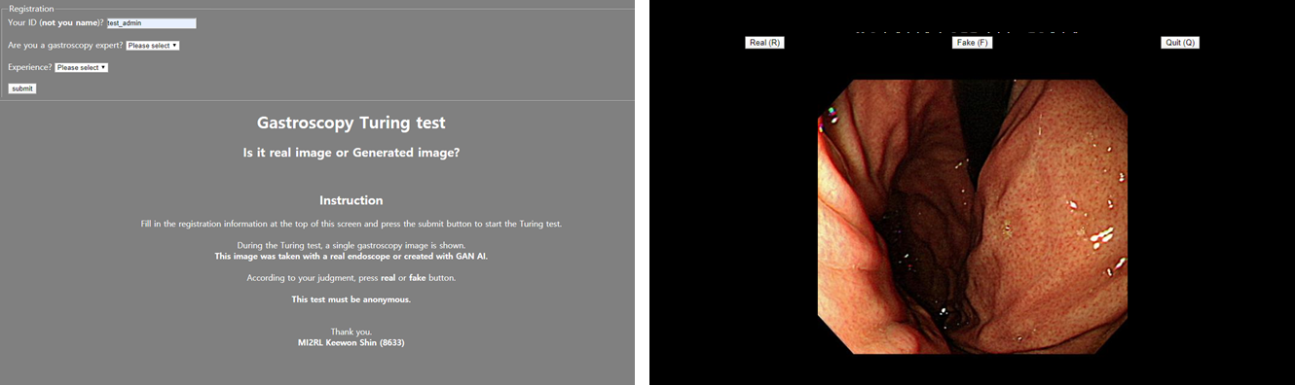
**

**Figure S2.** A visual Turing test website for gastroscopy images, synthetic images generated by our PGGAN model, and real images were randomly mixed and displayed on the website. Nineteen endoscopists independently determined whether each image was real or fake.
